# Applying a gender lens to understand pathways through care for acutely ill young children in Kenyan urban informal settlements

**DOI:** 10.1186/s12939-020-01349-3

**Published:** 2021-01-06

**Authors:** Kui Muraya, Michael Ogutu, Mercy Mwadhi, Jennifer Mikusa, Maureen Okinyi, Charity Magawi, Scholastica Zakayo, Rita Njeru, Sarma Haribondhu, Md. Fakhar Uddin, Vicki Marsh, Judd L. Walson, James Berkley, Sassy Molyneux

**Affiliations:** 1 KEMRI-Wellcome Trust Research Programme, P.O. Box 43640-00100, Nairobi, Kenya; 2 KEMRI-Wellcome Trust Research Programme, P.O. Box 230-80108, Kilifi, Kenya; 3 Nutrition and Clinical Services Division, icddr,b, Mohakhali, Dhaka, 1212 Bangladesh; 4 Research School of Population Health, Australian National University, Acton, ACT 2601 Australia; 5 Centre for Tropical Medicine & Global Health, Nuffield Department of Medicine, University of Oxford, Old Road Campus, Headington, Oxford, OX3 7BN UK; 6 Department of Global Health, University of Washington, 1510 San Juan Rd NE, Box 357965, Seattle, WA 98195-7965 USA

**Keywords:** Gender, Child health, Urban health, Pathways to care, Urban informal settlements

## Abstract

**Background:**

In many African settings, gender strongly influences household treatment-seeking and decision-making for childhood illnesses. While mothers are often the primary engagers with health facilities, their independence in illness-related decisions is shaped by various factors. Drawing on a gender lens, we explored treatment-seeking pathways pre- and post-hospital admission for acutely ill young children living in low income settlements in Nairobi, Kenya; and the gendered impact of child illness both at the household and health system level.

**Methods:**

Household members of 22 children admitted to a public hospital were interviewed in their homes several times post hospital discharge. In-depth interviews covered the child’s household situation, health and illness; and the family’s treatment-seeking choices and experiences. Children were selected from an observational cohort established by the Childhood Acute Illness and Nutrition (CHAIN) Network.

**Results:**

Treatment-seeking pathways were often long and complex, with mothers playing the key role in caring for their children and in treatment decision-making. Facing many anxieties and dilemmas, mothers often consulted with significant influencers - primarily women - particularly where illnesses were prolonged or complex. In contrast to observations in rural African contexts, fathers were less prominent as influencers than (often female) neighbours, grandparents and other relatives. Mothers were sometimes blamed for their child’s condition at home and at health facilities. Children’s illness episode and associated treatment-seeking had significant gendered socio-economic consequences for households, including through mothers having to take substantial time off work, reduce their working hours and income, or even losing their jobs.

**Conclusion:**

Women in urban low-income settings are disproportionately impacted by acute child illness and the related treatment-seeking and recovery process. The range of interventions needed to support mothers as they navigate their way through children’s illnesses and recovery include: deliberate engagement of men in child health to counteract the dominant perception of child health and care as a ‘female-domain’; targeted economic strategies such as cash transfers to safeguard the most vulnerable women and households, combined with more robust labour policies to protect affected women; as well as implementing strategies at the health system level to improve interactions between health workers and community members.

## Background

Gender - the socially constructed roles, behaviours and resulting relationships that a given society considers appropriate for men and women [1, 2] - plays an important role in child health. In many African households, women are the primary carers of young children but are less likely than men to be the principal resource providers or primarily in control of available household resources [3–5]. In rural African settings in particular, gender and family relations feature heavily in treatment decision-making processes for childhood illness [5–10]. Studies have shown that women are often held ultimately responsible for the health of their children, but many household members and other social network members can be involved in treatment-seeking actions [6, 7, 9, 11–14].

Whether or not mothers make independent decisions regarding treatment of child illnesses is determined by a range of inter-related factors including the nature and perceived severity of illness, who is perceived to ‘own’ the child, perceived cause of illness and intra-household roles and relations [5, 9, 10]. Studies in rural African contexts have also shown that a child’s father and/or other elders in the household are likely to have to be consulted for illnesses regarded as complex, serious or life-threatening. In some cases, the child’s father has definitive authority over the type of treatment sought [5, 9, 10]. In other cases, and particularly in extended homesteads, household elders - both male and female - may have stronger authority than young fathers/husbands [5, 8]. This decision-making power among household seniors can be linked to their control over available resources, as well as to the authority and respect afforded to elder members of the family [9]. Given the above, treatment-seeking for child illness may not be at the discretion of the child’s mother, but rather is shaped by inter-personal relationships and cultural prescriptions. Whilst there is an extensive literature on gender relations and child treatment-seeking in rural Africa, these dynamics have rarely been explored in the urban context, and specifically in low-income settlements.

The number of urban residents worldwide is rapidly increasing with one in three of those living in low- and middle-income countries (LMICs) residing in urban informal settlements [15]. In sub-Saharan Africa, more than half of the urban population live in informal settlements (colloquially known as ‘slums’), and it is projected that this number will increase to 1.2 billion by the year 2050 [16]. In Kenya where this study was conducted, it is estimated that more than half of the urban population lives in such informal settlements, for example 56% of residents in the capital city Nairobi [16]. These populations are often characterized by a disproportionately higher burden of disease [17, 18] and limited access to health care [19, 20]. They also face a host of other challenges including severe financial constraints, inadequate access to water and good sanitation, poor housing conditions and poor livelihood opportunities [17, 19, 21]. According to the 2014 Kenya Demographic and Health Survey [22], the child mortality rate in urban slums was significantly higher than the national average, the urban average and the rural average. Similar higher figures were observed for total under-five mortality in urban slums compared to the national and urban average, indicating an urgent need for better understanding of treatment pathways and decision-making during child illnesses in these settings. Indeed, some authors have argued that informal settlement health should be a substantive topic of study distinct from urban health; as issues related to the former tend to subsumed in the latter, obscuring important particularities such as spatial and neighbourhood effects that play an important role in health [19].

In this paper, focusing on the household as the unit of analysis and drawing on a gender lens, we explore treatment-seeking pathways into, through and post hospital discharge for acutely ill young children living in low income settlements in Nairobi, Kenya. We are not aware of any other studies in Africa that have used the longitudinal qualitative approach that we have taken.

## Methods

This work was undertaken as part of a broader international, multi-disciplinary research network known as the Childhood Acute Illness and Nutrition (CHAIN) Network described elsewhere [23]. The CHAIN Network has nine sites in six countries within Africa and Asia, including Kenya. The overall aim of the CHAIN Network is to identify the biological mechanisms and the socio-economic factors that determine a child’s risk of mortality in the 6 months following presentation to medical care with an acute illness [23]. As part of this broader goal, a qualitative social science sub-study was undertaken in Nairobi in urban Kenya.

### Study sites and data collection

This qualitative longitudinal study was conducted in two urban informal settlements of Nairobi County. Twenty-two families of acutely ill children aged 2–23 months with varying nutritional status who had been admitted to the study hospital and enrolled in the CHAIN observational cohort [23] were followed up over an 18-month period post-hospital discharge. Nutritional status is known to have a major influence on child survival, with undernutrition having a synergistic effect with acute illness [24, 25]. The initial intention was to purposively select equal numbers of families across three strata of nutritional status: severe wasting or kwashiorkor (SWK), moderate wasting (MW) and no wasting (NW); with varying socio-economic vulnerability (based on prospectively collected data on maternal education, household access to financial and social resources and household size). In practice, extended public health worker strikes and ensuing hospital closures significantly impacted our recruitment and led to us selecting families based on residence in two low-income settlements (Kibera and Mathare). We finally included eight, eleven and three households of children with SWK, MW and NW respectively, of varying socio-economic vulnerability. Kibera - the largest urban informal settlment in East Africa - was selected based on its proximity to the study hospital. The majority (18/22) of families in this study resided in Kibera. According to data collected by the Africa Population Health Research Centre (APHRC), as of 2012 Kibera had an infant mortality rate of 33.2 deaths per 1000 live births, post neonatal mortality rate of 45.3 deaths per 1000 live births and a total under-five mortality rate of 78.5 deaths per 1000 live births [17]. The remaining four families resided in Mathare, a populous informal settlement consisting of a constellation of slums, that is located a few kilometers north east of Nairobi’s central business district [26]. In addition to being the second largest informal settlement in Kenya, Mathare was selected based on previous existing relationships with key community stakeholders and gatekeepers, which helped to ease our entry into the community to undertake the household follow-ups.

Each household was visited 2–3 times during the follow-up period, totaling 58 visits across the 22 households. For ethical reasons, household follow-ups (beyond a standard condolence visit) were discontinued in three of the households where the target child died following our initial visits. In-depth interviews conducted during the household visits were primarily with the children’s main carers and other family members, and covered a broad range of topics including: child health and nutrition; the child’s illness trajectory and related treatment-seeking and decision-making; experiences with the admitting hospital and the health system more broadly; as well as challenges faced during the child’s illness episode and coping strategies. All interviews were audio-recorded. Written informed consent was obtained from all participants in the initial household visit with verbal consent obtained in all subsequent visits to continue in the study. Non-participant observations were also conducted at the admitting hospital and at household level to give a sense of living conditions and family dynamics, community relations, as well as experiences and interactions at the hospital.

### Data analysis

Data were analysed using a modified framework approach. This entailed: extensive familiarization with the data (‘immersion’ in the data by reading and re-reading of transcripts, listening to audio-recordings and reading field notes); condensing the data into detailed summary sheets per household across visits and subsequently developing and refining a master summary for all households across all visits; consultatively developing a coding framework based on preliminary emergent themes and the study objectives; and coding the entire dataset into NVivo software to search for broader emergent themes. Comparison tables were also developed to identify patterns for example based on child nutritional status or household structure and headship; as well as ‘rich stories’ for each household to ensure that the broader narrative of each household was preserved.

Concurrently with the framework approach, gender analysis was undertaken drawing on the gender framework by Morgan et al. [1] (Table 1). According to this framework, gender analysis can be incorporated into health systems research *content, process* and *outcomes*. For our analysis, we focused on the *content* and specifically examined gender relations at household and community level in the context of childhood acute illness, including exploring: who has what (access to resources); who does what (the division of labour and everyday practices); how values are defined (social norms) and who decides (rules and decision-making) [1]. We also reviewed our data for any illustrations of how power is negotiated and changed in households and communities.

**Table 1:** Gender as a power relation and driver of inequality

What constitutes gendered power relations	
Who has what?	Access to resources (education, information, skills, income, employment, services, benefits, time, space, social capital etc.)
Who does what?	Division of labour within and beyond the household and everyday practices
How are values defined?	Social norms, ideologies, beliefs and perceptions
Who decides?	Rules and decision-making (both formal and informal)
How power is negotiated and changed	
Individual/People	Critical consciousness, acknowledgement/lack of acknowledgment, agency/apathy, interests, historical and lived experiences, resistance or violence
Structural/Environment	Legal and policy status, institutionalisation within planning and programs, funding, accountability mechanisms

## Results

This section begins by presenting an overview of the study households (HHs) followed by a description of the treatment pathways undertaken during childhood illness. For this study we defined households as family members who lived together in the same home for most of the time. Findings are then presented to illustrate how gender manifested itself throughout the treatment pathway for acute child illness; paying particular focus to the gendered impact of the illness on the household, and gendered experience of the health system. These findings are then considered in relation to the Morgan et al. [1] framework in the discussion section.

### Household characteristics

Table 2 below summarizes the types of households (HH) involved in this study, including income type and the family size and structure at our *initial* visit to the household. It is, however, important to note that any simple categorization of the households is problematic, as dynamics sometimes changed - including in important gendered ways - over the course of our visits. Indeed, a child’s illness sometimes fed into and was influenced by these changes over time. For example, some mothers who previously held regular paid jobs or ran businesses gave up their income-generating activities to focus on care of their ailing child; and others - in our later visits - started working (away from the home) more regularly, and put the child in paid day care, once their health stabilized. This fluidity of household dynamics cannot be captured in a single table but will be illustrated further in the findings below.

**Table 2:** Summary characteristics of the study households (HH)

Code	Nutritional status	About main caregiver				About family/household			
		Age	Relation to index child (IC)	Education level	Job type	Family Type	No. of income earners	Main income earner’s job type	No. of people in the family
1	SWK	22	Mother	Secondary	Casual	Extended (single mum living in natal home)	1	Regular	6
2		33	Mother	Primary	Self employed	Nuclear	1	Self employed	7
3		19	Mother	Primary	Unemployed	Nuclear	1	Casual	3
4		29	Mother	Primary	Unemployed	Nuclear	1	Casual	3
5		24	Mother	Primary	Casual	Extended	1	Casual	6
6		22	Aunt	Secondary	Unemployed	Extended (single mum living in natal home)	4	Regular	19
7		22	Mother	Primary	Unemployed	Nuclear	1	Casual	4
8		20	Mother	Primary	Unemployed	Nuclear	1	Casual	3
9	MW	19	Mother	Secondary	Unemployed	Extended (single mum living in natal home)	2	Regular	7
10		25	Mother	Diploma	Unemployed	Single parent	1	Unemployed (mother relies on her siblings)	2
11		19	Mother	Primary	Unemployed	Nuclear	1	Casual	4
12		30	Mother	Secondary	Unemployed	Nuclear	1	Regular	6
13		28	Mother	Primary	Unemployed	Polygamous Extended	1	Casual	8
14		30	Mother	Primary	Unemployed	Nuclear	1	Casual	4
15		19	Aunt	Diploma	Unemployed	Extended	2	Regular	5
16		43	Grandmother	Primary	Casual	Extended (Single mum living in natal home)	1	Casual	3
17		26	Mother	Primary	Casual	Nuclear	2	Casual	8
18		20	Mother	Primary	Unemployed	Extended (Single mum living with maternal aunt)	2	Self employed	5
19		22	Mother	Primary	Casual	Polygamous Nuclear	1	Casual	7
20	NW	27	Mother	Primary	Unemployed	Nuclear	1	Casual	3
21		28	Mother	Primary	Unemployed	Nuclear	1	Regular	4
22		–	Mother	Primary	Casual	Nuclear	2	Casual	4

There was a mix of nuclear and extended family structures, with two households categorised as polygamous. In both polygamous homes, the target child’s mother lived in the urban informal settlement with the child’s father and her own children, while her co-wife/wives lived in their rural home. In Table 2 above, the ‘number of people in the family’ only includes those who lived in the urban residence. Most of the main caregivers in this study were the target child’s mother but two children were primarily cared for by their maternal aunts and a third child by his maternal grandmother. In these three latter cases, the children’s mothers were single parents who still lived in their natal homes, including one who was a secondary school student. All the main carers had some level of primary formal schooling, with six carers having post-primary education (four secondary school and two diploma-level). Although most of the carers categorized themselves as ‘unemployed’ or ‘not working’, it is important to note that some of them earned minimal irregular income from activities such as occasionally braiding neighbours’ hair.

### Treatment pathways

In this study, treatment-seeking pathways were often long and complex, with carers either moving between or simultaneously using, biomedical and alternative treatments depending on the perceived cause of illness. This applied both pre- and post-hospital discharge. None of our respondents discussed using alternative treatments while admitted in hospital, although we know anecdotally from the study hospital staff that this does sometimes happen.

At the onset of illness symptoms, the majority of carers (16 HHs) first visited local government or non-governmental health centres within their home area to seek initial treatment for their children. Among the remaining six families, four first visited local chemists, one went directly to a herbalist, and another resorted to ‘home-treatment’ using left over medicine in the household. It is possible that health centres, especially non-governmental ones, were a preferred first point of treatment action as services there are offered free or at a highly subsidized price. These initial treatment actions were then followed by a myriad of other responses before children finally ended up at the admitting hospital. Responses included: repeated visits to the same or other local health centre(s); visiting a higher level faith-based or government facility; ‘waiting’ to see if the condition would resolve on its own; buying additional or different medication from shops and chemists; visiting a range of herbalists and traditional healers; and seeking ‘spiritual intervention’ from religious leaders. The quote below shows the range of actions that carers took in attempting to restore their child’s health:

*“When the child first fell sick with chicken pox, I did not immediately take him to hospital … it was a Saturday and all the free clinics were shut and I also didn’t have money. So I took my brother’s medicine [which he had been using when he had chicken pox] and applied it on the child’s body … the condition was still the same, so I took him to a private clinic … they carried out tests … and gave him medicine for malaria, cough and diarrhoea. But he still did not recover...I also tried treatment from [several] herbalists because I was not seeing any change … he again fell sick … I bought medicine … from a chemist … but still the cough persisted. Then I decided to go to an NGO (non-governmental) facility so they could carry out tests on his chest. It was at this place that I was told the child requires hospital admission* …” (IC mother, HH 009, visit 1).

**Fig. 1:**
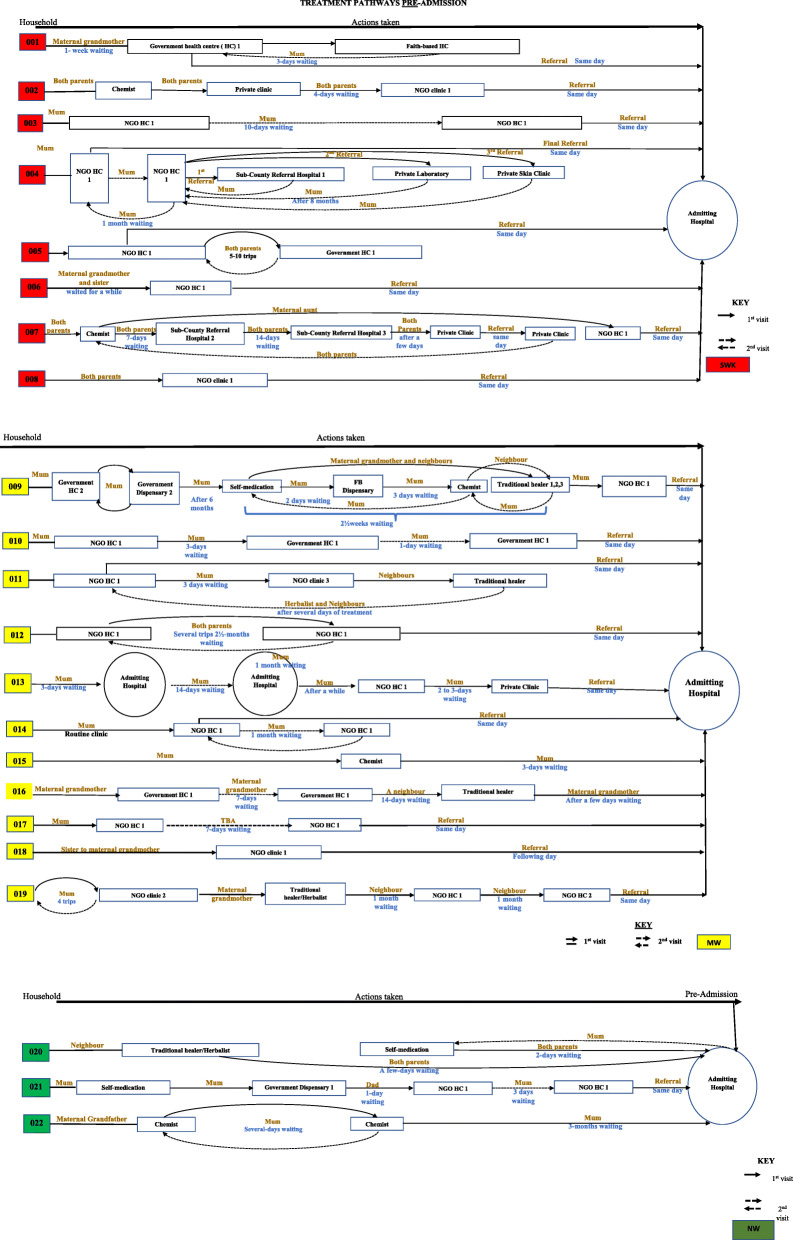
Pre-admission treatment pathways by nutritional status

Figure 1 below, illustrates the range of treatment actions (pre-admission) that were taken by families throughout the child’s illness trajectory, including ‘waiting time’ before they eventually ended up at the study hospital, which was usually by referral from a lower level health facility. Similar dynamics were observed post-hospital admission, although to a lesser extent and with generally much fewer treatment actions as many of the children’s health was reported by families to have stabilized post-admission. Only one child (HH012) was readmitted (twice) to the study hospital post-discharge. It is notable that the CHAIN cohort activities also involved regular follow-ups back up at the admitting hospital for all cohort children, which may have replaced other potential treatment-seeking actions.

### Gendered decision-making and treatment-seeking for child illness

As is the case in many other African settings, in this study childcare and health was predominantly a female domain, with women being the main engagers with the health system and - in some cases - supplementing the household income through various (mostly casual) income-generating activities. Men were largely absent in the day-to-day care of the children other than as financial providers for treatment-seeking. Mothers were also the main decision-makers in relation to child health and treatment-seeking but there were other, primarily female, key influencers throughout the pathway. These included female neighbours and friends, grandmothers and other female relatives such as maternal aunts. The box story below illustrates the complex treatment-seeking pathways during child illness and their gendered nature. These gendered nuances are discussed in more detail below.

**Table:** 

**HH 19** Mercy* is aged 1 year, 5 months and lives in Slum X with her 22-year-old mother, her father, and three older siblings. Her mother is the youngest of 4 wives (two other wives are deceased), having been ‘inherited’ by her late sister’s husband. They live in a single-roomed house made of corrugated iron sheet with an earth floor. Mercy’s mother has standard six-level of formal education and is unemployed, although sometimes she does casual jobs washing clothes. She primarily relies on her husband (Mercy’s dad) - who works as a casual labourer - for financial provision, and sometimes gets help from her own mother who lives in the village, or her cousin who lives in Nairobi. Mercy’s father also supports his extended family. Mercy was born of good health up until seven months of age when she started falling sick with diarrhoea and poor appetite. Initially, her mother thought the illness was due to teething (*vitu za mdomo*) and assumed it would go away on its own. When Mercy’s condition did not improve, the mother first sought treatment at a nearby NGO health facility. She made four visits to this facility and was given the same treatment. With no signs of improvement, she decided to seek advice from her own mother (Mercy’s grandmother) who informed her that she might have ‘crossed paths with a bad person’. The grandmother therefore advised her to go and collect rubbish (waste paper, pieces of plastics, sticks, etc.) from a crossroad somewhere and burn them at home; after which she was to apply part of the ash on the child’s forehead, and then mix the rest with water for Mercy to drink. Thereafter, Mercy’s condition improved for about two weeks but then deteriorated further. After a month of ‘waiting and seeing’, Mercy’s mum consulted one of her neighbours who is also a friend. The neighbour referred her to her landlady who is a community health volunteer (CHV) at another NGO health facility. The landlady then referred them to that NGO facility where Mercy received medication for cough and chest pain, before being referred to a nearby sub-county hospital. However, Mercy’s mum did not follow through with the referral since she did not know where the hospital was located, and the father was unwilling to provide the required financial support for the treatment. Mercy’s condition further deteriorated. About a month later, Mercy choked on porridge while being fed and the mother sought for help from another neighbour. The neighbour then suggested they take her to *japollo,* a man in the area known for praying for the sick. However, the neighbour who had initially referred her to the CHV, suggested they take the child to a nearby faith-based health facility. It was at this clinic where Mercy was referred to the study hospital for admission. She was admitted for nine days and treated for pneumonia and pulmonary TB. The mother was given referrals to health centres in their area of residence for ongoing TB treatment. Mercy was also enrolled into a supplementary nutrition programme. However, Mercy’s mother has found it difficult to implement the medical and nutritional advice given due to financial constraints.	

As illustrated in Fig. 1, mothers played a key role in deciding on treatment action and had significant autonomy to do so even when the child’s condition was perceived to be serious. Nonetheless, there were other - predominantly female - individuals who influenced the process including: the target child’s grandmother; other relatives such as the mothers’ sisters and aunts; neighbours and friends; and traditional and religious healers. Mothers’ independent ability to decide on child treatment was linked to their caring role, with the children’s fathers usually being informed for the purposes of providing the required financial support rather than being consulted or making the decision. Indeed, occasionally when the mother felt that the condition was not ‘sufficiently serious’ they would ignore the fathers’ suggestions to take the child to hospital until they deemed it necessary as illustrated below:

*“After around the fifth month [of illness], the father told me take this child to hospital. But I did not listen [because I did not think it was serious]. Instead … I would take him to the sister [chemist] nearby, depending on how I gauge his condition. [Initially] I did not think it was serious … [But when I saw it was] I immediately rushed the child to the [study] hospital. I didn’t even call the father to tell him where I was going. I asked another woman [neighbour] to inform him that I had gone to the hospital”.* (IC mother HH 022 visit 1).

The child’s mother was the primary decision-maker, often in consultation with others in 12/22 households. In other households (6/22), decisions were made consultatively between both parents and consensus reached on the best way forward. In fewer cases (4/22) the father was the primary decision-maker on treatment to be undertaken, sometimes pegged on their being the primary or sole financial provider. Although sometimes mothers felt restricted in their ability to take immediate action due to financial reliance on their spouses, as illustrated in the quote below, most stated having relatively easy access to the household financial resources as and when needed and a ‘cooperative dynamic’ in the interest of the child’s wellbeing. It was only when there was significant marital disharmony (for example in Mercy’s case story above, and in one other example in our households) that we observed an unwillingness by the father to provide resources for the child’s care.

“*… now I can buy [medication]. If I have money I will just go and buy. In the past you know I had to wait for the father to get money then I get it from him. But now [that I have a job] if I have money I will just go and buy for the child what I want.”* (IC mum HH 020 visit 2).

As earlier stated, older women (usually grandmothers or sometimes senior women in the community) acted as advisors and gave guidance to younger mothers on treatment-seeking. This was often the case where the child’s mother was a sole parent and still residing in her natal home. These mothers were also relatively young. In such situations, the older women played a significant role in the general care of the child, decision making during illness and financial provision to access treatment. The illustrations below show the important role played by these senior women.

*“… you know she’s my first child. Sometimes I don’t know she is sick, she [grandmother] is the one who tells me the child is sick and that I should take her to hospital. I don’t usually know … so when she comes [home from work] I ask her, she tells me that she is sick, and I should take her to hospital the next day.”* (IC mother HH 001 visit 1).

*“… I don’t know which drug they gave him [the child] at the herbalist’s … Because I already tried all kinds of medicine. It’s mum [child’s grandmother] who advised that we stop giving him the medicines [and instead use herbal treatment] since they made him weak … It’s at that point when they gave him the herbs …”* (IC mother HH 009 visit 1).

Nonetheless, family arrangements where young single mothers lived in their maternal homes were not without challenges, especially when the child’s mother and grandmother disagreed on the course of treatment. In one home for example, the grandmother - who was the child’s primary carer as the mother was in secondary school - believed that the child ‘s illness was due to the ‘evil eye’ (*kutupiwa*). Upon the advice of her neighbours, she started administering herbal medication which resulted in the child’s health deteriorating further. The young mother on the other hand, perceived the cause of illness to be biomedical. This resulted in significant tension in the household, with the young mother ultimately taking the child to the (study) hospital against her mother’s wishes.

Seeking guidance and advice from older women was not only for young single mothers. Generally, younger parents in this study would seek guidance from older women in the community as they regarded them as knowledgeable, trustworthy and wise; and able to offer appropriate advice in times of crisis with a child’s health. Below a father narrates how he and his wife trusted the older women’s advice:

*“There are these mothers … older mothers, those that understand these things, you know when you are young, you have to ask those older than you how things are done. So, when they come with their suggestions you will have to listen... you must listen, if they advise you, you will have to listen. So that’s how we do it.”* (IC father HH 020 visit 1).

In some instances, married women would also contact via phone their own mothers and/or mothers-in-law who lived in the rural area to seek guidance and advice regarding the child’s condition and suitable treatment, as was the case in Mercy’s story above (Box 1).

Female neighbours were another very influential group of people throughout the child’s illness trajectory. They were sometimes the first point of contact when a mother was unsure about their child’s symptoms; they gave opinions on perceived cause of illness, and suggested appropriate treatment action, which in many cases mothers adhered to. Additionally, they acted as a crucial support system for the mother, for example by caring for other children when a parent had to stay in hospital with an admitted child. The quotes below illustrate the important influence of neighbours:

*“They [neighbours] were saying the child has been looked at with the evil eye (kutupiwa) or bewitched, so I didn’t understand that … they said that there is a well-known woman in this area who cures the evil eye. So, I took the child there … she mixed salad (i.e. vegetable cooking oil) ...I had gone with the salad, they [neighbours] had told me that I should go with the salad …”* (IC mother HH 009, visit 1).

*“… when the child started getting sick, [neighbours] told me it’s mambo ya ukoo (family issues) …*. *they started telling me that maybe the [father’s] family went to a witchdoctor so that I can give them the baby …. So, they said I should take a cold shower with the baby, and when the droplets of water splash on her, [the evil spirit] will leave the child’s body …”* (IC mother, HH001, visit 1).

### Gendered impact of illness on the household

In nearly all of the households, the child’s illness episode had a significant impact on the household, and this was sometimes gendered.

In particular, some mothers narrated how they had to leave employment (voluntarily or dismissed), reduce working hours or close their businesses to care of their sick children. This was especially the case where illness was prolonged, or where - due to the child’s dire condition - the mothers wanted to be present after discharge from hospital to ensure the child fully recovered. This in turn resulted in reduced earnings for both the mother and the household more broadly. It also increased mothers’ financial reliance on others such as husbands or grandmothers. Below, one mother explains how she chose to shut down her business to care for the child, while another single mother was fired from her place of work for long absences while the child was admitted (this latter mother had resumed full time employment by our third visit to the household) .

*“I closed the shop because I wanted to care for the baby. You see when the child was admitted, I couldn’t continue running the shop … and the child wasn’t getting better, so I saw there was no point...I saw if I keep the shop open, with this child’s condition, I will suffer. The way I put in so much effort to care for this child? You know even God helps those who help themselves. You can’t leave the child here [at home] or take the child to day care and go to work [yet the child is sick].”* (IC mother HH 012, visit 2).

*“When the child fell sick the restaurant owner [where I was working], told me to stop working. I did not go to work for a long time because we were admitted at the hospital. So, he said I should stop going to work.”* (IC mother HH 009, visit 1).

Another gendered impact of child illness was blame towards the mother by family members (as well as health workers, as returned to below). Mothers described sometimes being held accountable for their child’s poor health condition as they were the primary carers. There was an implicit expectation that women should pay attention to, and take good care of, their children including taking necessary precautions to ensure that they do not fall sick. As such, when child illness occurred, it was sometimes perceived as indicating sub-optimal care from the mother. This would also sometimes result in marital tension within the home as illustrated below:

*“It happens [the blame] … sometimes we used to disagree since the father always claims that I do not cook proper food for the child [and that is why she fell sick], but the problem is when I cook the child doesn’t eat [i.e. child is a reported poor feeder]. At times I ask him to feed the baby himself [so he can see], he will try and claim that he does not know how to feed the child, so it is my responsibility to feed her.”* (IC mother HH 021 Visit 2).

Furthermore, most of the mothers we interacted with had to juggle between general care of their home and other children, as well as giving dedicated attention to the ailing child especially during hospital admission, which was sometimes challenging. Mothers often had to leave their other children in the care of female neighbours and other relatives. It was especially difficult when the other children were also young, for example in the illustration below where one twin was admitted to hospital and the other had to be left at home in the care of others. In this case, the mother would spend the entire day in hospital with the sick twin and then leave him in the care of her sister (the child’s aunt) at night, so she could return home and breastfeed the other twin for fear that she would also fall sick.

*I used to come back in the evenings and care for the twin. During the day, I used to make her porridge in the morning and leave her with my (female) cousin, then go to the hospital and come back at six in the evening … My sister is the one who used to spend the night at the hospital. I used to sleep at home, because of the twin sister since I did not want her to stop breastfeeding and also start disturbing me [also fall sick].”* (IC mother HH 005 visit 1).

### Gendered experience of the health system

As earlier indicated, women were the primary engagers with the health system throughout the child’s illness trajectory (although some male relatives were observed in the wards during visiting hours at the study hospital). As such, women were the ones who dealt with the ‘day-to-day’ challenges of interacting with a less-than-optimal health system. This manifested in different ways including challenging interactions between mothers and healthcare workers or hospital support staff, particularly around poor communication. Caregivers reported especially difficult interactions with female nurses, often making a clear distinction between male nurses (as being ‘nicer’) compared to their female counterparts. Some respondents described female nurses as rude, uncaring, unfriendly and disrespectful towards the mothers; including sometimes blaming and reprimanding the mothers for their children’s deteriorating health. Below, a mother explains what she deemed as an uncaring attitude from nurses in the admitting hospital:

*“You call her [the ward nurse] to come and check on the child but she is not concerned. She just sits there at her desk. You go back thrice. You wait for her she still doesn’t come, so you go and call her again and she tells you if you are in a hurry, you can treat the child yourself. Now you wonder how you should start treating the child yourself for the illness that made you take the child there [to hospital] in the first place?*” (IC mother HH 012 visit 3).

In another case, a mother opted not to follow the prescribed treatment for her severely undernourished child while admitted in hospital, due to challenging relations between one of the health workers (nutritionist) and the mothers in the ward.

*“[The child was not removed from the nutrition programme] I am the one who stopped going … they were two nutritionists, one was bad and one was good … the bad one, she would shout at the mothers in the ward … you express breastmilk when she is there and it doesn’t come out, but she says you have enough milk … the women in the wards were complaining because she would refuse to give us [supplementary] milk for the children...so when she was the one on duty I wouldn’t go [to collect the nutrition supplements].”* (IC mother HH 004 visit 1).

These negative interactions also had an impact on continuing care post-hospital admissions, resulting in mothers sometimes discontinuing recommended care. For instance, one mother whose undernourished child had been enrolled in a nutrition programme in a local health facility following discharge, halted her involvement due to poor communication from the nurses as indicated below:

*“I decided to remove the child [from the nutrition programme] because the child was always sick … when the child falls sick they [nurses] would reprimand me whenever I’d go to collect the food supplements … they would say that I am selling the flour … and I don’t give the child the porridge … so I decided to stop going …”* (IC mother HH 011 visit 2).

Other challenges that mothers reported experiencing in their interaction with the health system included: long waiting times even when children were in dire condition and mothers had been referred upward from other facilities; sharing beds with other mothers and their children while admitted in hospital, which also raised concerns around cross-infection; poor and inadequate hospital facilities including power and water shortages at the admitting hospital which sometimes increased their overall indirect expenses as highlighted below:

*“… There was a water shortage [while we were admitted at hospital]. One could not even get a place to relieve themselves...I had to go and pay for a toilet outside … because the toilets were full of waste, you couldn’t use them … [when] there is no water it becomes a real problem.”* (IC mother HH 012 visit 2).

Despite these challenges, particularly while admitted in hospital, mothers found ways to support each other materially and socially, for example through sharing items such as diapers or taking care of each other’s children when one needed to step out.

*“If you have stepped out of the ward and it is time to eat … the others [mothers] would ensure you do not miss food … or if you miss they would share their food. Also, those who were being newly admitted from home and didn’t have a basin, slippers, soap or pampers [as sometimes the admission was unexpected], we would share”* (IC mother HH 006 visit 1).

They also comforted and provided each other with emotional support while in the wards, especially when one was experiencing difficulties related to their child’s health condition.

## Discussion

Twenty-two families of children admitted in the study hospital with varying nutritional status were individually followed up repeatedly for a period of 6 months post-discharge (totalling 18 months of household follow up visits). The findings from this study illustrate that pathways through care for acutely ill young children in Kenyan urban informal settlements are both complex and highly gendered with women taking a predominant role. Drawing on the Morgan et al. [1] gender analysis framework, Table 3 below summarizes the gendered nature of household treatment-seeking, highlighting specific intersections of gender and age, as has also been observed in other African contexts [6, 27–29].

**Table 3:** Summary of the gendered nature of household treatment-seeking

What constitutes gendered power relations			How is power negotiated and changed?		
Who has what	Who does what	Who decides	How are values defined	Household level	Health system level
Men (and to a lesser extent senior women), had more direct access to financial resources by virtue of being the income-earners.	Women (mothers, grandmothers and female neighbours) were the primary carers of children, decision-makers and engagers with the health system during child illness. Female relatives and neighbours play a key role in influencing perception of the child’s illness cause and advising on ‘suitable’ treatment options. Where present, men and senior woman such as grandmothers (where they resided in the household), were the primary financial providers including for treatment-seeking.	Mothers were the primary decision-makers for treatment-seeking during child illness, largely informing husbands (where present) for financial provision to seek treatment. Where senior women resided in the household (HH), they also played a key authoritative or advisory role in decisions around child health, illness and treatment-seeking. In fewer cases, there were consultations and joint decisions between the fathers and mothers	As is typical of the study context, childcare roles and responsibilities were gendered with women being primarily responsible, and caregiving perceived as a female domain. When children were unwell, some mothers were blamed for failing to meet these responsibilities. Men’s roles and responsibilities were primarily centred on providing funds. Older women both within and outside the HH were regarded as wise and considered to have knowledge, and experience in matters of child health. Consequently, they acted as advisors and provided guidance to younger parents.	There appeared to be ‘cooperation’ between spouses when it came to child health; with mothers stating easy access to HH financial resources for treatment-seeking; despite predominantly relying on husbands for financial provision. Two exceptions were noted, both of which had marital disharmony. In both cases, the mothers drew on extended family support. Most mothers had autonomy to decide where to seek treatment for their ill children with limited or no consultation. As younger wives, the two mothers in polygamous unions had to navigate challenging relationships with their older co-wife/ves (who lived elsewhere) and reduced financial support from their spouses.	There were more evident power hierarchies within the health system; particularly between female nurses and mothers. Female nurses were described as often exerting dominant power over mothers resulting in poor interactions and compromising childcare. This was both in the outpatient and inpatient settings but more pronounced in the latter. Mothers on the other hand negotiated this by exercising subtle agency e.g. by; drawing on each other for support, refusing to attend clinics on days when certain (perceived unfriendly) health workers were on duty, or expressly ‘demanding’ attention when they felt their children were neglected.

In this study - as in many other African contexts - women were the primary caregivers and engagers with the health system during times of childhood illness. In most households, they had significant autonomy to make decisions related to treatment actions - even when the condition was perceived as serious - only informing their spouses (where present) if they required financial provision. These findings support previous work undertaken in Kenya by Amuyunzu-Nyamongo and Nyamongo (2006) investigating health seeking behaviours of mothers of under-five-year-old children in urban informal settlements [30]. In the 2006 study, they found that mothers were able to make decisions about treatment of ill children, with the primary determining factor being the perceived nature and severity of the illness. These urban dynamics, however, contrast sharply from observations in rural African settings including Kenya; where mothers, especially younger mothers are observed as having to consult, seek permission and/or negotiate with various other household members including spouses and elders before taking treatment action. This was especially required where child illness was perceived as serious or necessitating treatment outside of the community [5, 9, 10, 31, 32]. We are unaware of any systematic research that has been conducted to examine and understand this potentially important difference between rural and urban settings; but we posit that it could partly be attributed to varying family structures (often larger extended families in rural areas compared to the smaller nuclear units in urban settings), as well as more prevalent - and higher adherence to - ‘traditional’ norms around household headship, hierarchies and decision-making authority in rural areas.

As observed in other African settings, in this study senior women (whether within the immediate household, extended family or broader outside community) were often deferred to during child illness as wise, experienced and important advisors who are knowledgeable on child health. They also sometimes contributed financially even where they did not live in the same home, for example in the case story of Mercy. The important role of senior women in child health is well-documented in many African settings including Kenya, Tanzania, Senegal, Gambia, Malawi and Zimbabwe [5–7, 27–29, 31, 33]. In Tanzania for example, Muro et al. (2017) found that young parents consulted with older community women during acute child illness, and if they all agreed that the specific illness was malaria, then the younger parents would administer anti-malarial drugs [33]. In Malawi, Bezner-Kerr and colleagues (2008) found that paternal grandmothers have a powerful and multifaceted role within the extended family in terms of childcare and health with their ideas sometimes conflicting with the dominant biomedical views [6]. It is, however, important to note that in our study, this dynamic between younger parents and older women largely entailed consultation and seeking advice or guidance, rather than permission. This is in contrast to some studies in sub-Saharan Africa [5–7] and other low-and middle-income contexts such as India and Vietnam [34, 35] where senior women (in the home) were the decision-makers, with younger women having to seek approval from them before taking treatment action. Neighbours and peer mothers were also observed as playing a very dominant role in supporting and advising mothers during child illness. This entailed advising on the perceived cause of illness and subsequent ‘appropriate’ treatment; as well as being a crucial support system for the mother financially and in terms of moral support including helping with the care of other children when a mother needed to attend to the ailing child. The role of peer mothers as ‘advisors’ concurs with work undertaken by the first author in rural Coastal Kenya [31, 36]; as well as Shaw et al.’s (2016) Ethiopian study which showed that women would consult female neighbours with children in trying to understand their own child’s disease and available treatment options [37].

Another area that was overtly gendered with regards to a child’s illness episode was in relation to its impact on a household. Although the entire broader household was affected in one way or the other, our study findings suggest that mothers were disproportionately affected as a consequence of gendered roles and responsibilities around childcare. This included working mothers either voluntarily or involuntarily having to quit their jobs, reduce working hours, or close down businesses to care for the sick child. This gendered impact of illness as a result of women’s role as the primary child carers also manifested in the form of blame and stigma towards the mother when their child was not thriving. This was both at the household and health system level where mothers were sometimes blamed for ‘inadequate’ or ‘improper’ care of children resulting in their illness or poor health. In households, this had the potential to escalate into spousal conflict and familial disharmony. Blaming of women for poor child health is not unique to our study and has been observed in other African settings including in rural Kenya [36–38]. In rural coastal Kenya for example, Muraya et al. (2016) found that when children exhibited certain symptoms of ill health (a condition locally referred to as ‘*lugwizo’* but that would be categorized as undernutrition in biomedical terms), women were blamed for causing the disease due to poor child spacing. In this Coastal Kenya study, during discussions with respondents there was little consideration of the role that men played in a woman’s pregnancy frequency, or related gendered power dynamics and hierarchies that could potentially limit a woman’s ability to negotiate [36]. In another study in the same Coastal Kenya setting, Marsh and colleagues (2011) found that mothers were sometimes blamed by their families, particularly their sisters-in-law, when their children suffered from Sickle Cell Disease [38]. Similarly, Shaw et al. (2017) observed comparable dynamics in Ethiopia where women were blamed if the child’s illness was attributed to supernatural forces or environmental extremes, since it was considered their duty to ‘protect’ their children [37].

The gendered impact of a child’s illness episode was also demonstrated at the health systems level. As women were the main engagers with the health facilities (in their role as primary carers), they also ‘bore the brunt’ of challenges related to a less-than-than optimal health system. This included for example having to spend several hours waiting in queues at health facilities sometimes with severely ill children, and negative experiences at health facilities while admitted (for example due to lack of basic amenities such as water and electricity). Importantly, in our study there was a dominant emergent theme around power hierarchies illustrated in challenging interactions between mothers and both health workers and hospital support staff. In particular, female nurses were described as exercising dominant or authoritative *power over* [39] mothers; adversely impacting on mothers’ experience of the health system and highlighting the intersection of gender, professional and socio-economic status in shaping relationships and experiences. Mothers on the other hand exercised their agency and used more discretionary power such as the *power to* [39] act in certain ways, for example choosing when to or not to attend nutrition and health clinics depending on the existing relationships with, or perceived character of, specific health workers. Mothers - especially those admitted to hospital with their ill children - also to an extent drew on collective *power with others* [39], as a support mechanism and to help each other get through the challenges experienced within the health system; even if this did not necessarily translate to changes in the care and service received. Similar power dynamics have been observed in both Ethiopia [37] and Eastern Uganda [40] where mothers complained of less-than-optimal interactions with health workers including disrespectful behaviour and being reprimanded for their child’s poor health. In turn, mothers exercised their agency by not seeking services at particular health facilities or seeking alternative care altogether at places where they felt respected. As with our study, this sometimes had an impact on the child’s recovery process and their overall health.

### Limitation(s)

Although unintended, the key limitation of this study was the absence of men’s voices. All the fathers knew of our study and had no issue with us visiting their homes repeatedly. They were, however, present in only four of the 58 interviews. Most times we would find that the fathers were not at home when we visited; or if they were, they would welcome us and leave shortly after we started the discussions. Even in the four occasions where fathers were present, the mothers tended to do most of the talking with the former supplementing the information. The first author observed similar dynamics in her work on child health and nutrition in rural Kenya. Consequently, work has been planned that will draw on theories around masculinities and deliberately engage men in conversations on child health and nutrition, to ensure that no important voices are left out.

## Conclusion and recommendations

This study highlights gendered issues around decision-making and treatment-seeking during child acute illness and the gendered impact of an illness episode both at household and health system levels. All of these highlighted findings and dynamics operate within broader structural factors beyond the immediate household or the health system. Based on these findings we recommend:

- More deliberate engagement of men in child health to counteract the dominant perception of child health and care as a ‘female-domain’. In addition to systematic research in the area, this could include community-led strategies for example using ‘champions’ to raise awareness and encourage participation, and framing child health as ‘everyone’s business’. Additionally, primary healthcare facilities that are predominantly accessed by women and children and might be perceived as (or actually are) unfriendly towards men, should be organized in a manner that promotes and encourages male participation particularly as regards child health.
- The disproportionate economic impact of child illness on women such as through job loss suggests a need for more robust labour policies that ensure more family friendly work policies, and which specifically protect women from unfair dismissal for example for taking extended time off to care for ailing children. Unfortunately, the majority of women such as those in this study are employed in ‘precarious’ situations and on casual terms without formal contracts, which places them in very vulnerable positions. Various national advocacy groups such as the National Gender and Equality Commission, Kenya could work in collaboration with the Ministry of Labour, Social Security and Services and other stakeholders to ensure that policies and social welfare services are developed and implemented to protect such women. These longer-term policy-level approaches could be combined with other more immediate financial interventions such as unconditional cash transfers for the most vulnerable women and households, to buffer them during the child’s convalescence period. Despite some challenges in their implementation, in Kenya, government-led cash transfers using mobile money services have been shown to be appropriate and feasible during the current COVID-19 pandemic and subsequent loss of incomes for the most vulnerable households. Similar approaches could be adopted to support vulnerable families that experience income loss due to childhood illness.
- At the health system level, participatory and on-the-job training on communication skills and emotional competence could be implemented to help improve the working culture and interactions between health workers (and hospital support staff) and community members. A training programme developed over several decades and implemented across a range of African settings (as well as in Europe) has been shown to be very powerful in inspiring health workers to communicate more respectfully and effectively with their patients and colleagues (Haaland-ICARE model, 2020, available at https://connect.tghn.org). Elements of this course, which has at its core the building of self-awareness and reflection, could be built into continuous personal educational programmes, as well as incorporated earlier into health worker training. Additionally, at a broader level, it is important to ensure an adequately-staffed health workforce; which in turn avoids overworked and over-stretched health workers as this potentially also contributes to the less-than-optimal interactions with community members.

Our findings highlight the importance of applying a gender lens in understanding child illness, related treatment pathways and illness impact. It is evident from our study that a child’s illness episode impacts men and women differently, with women disproportionately ‘bearing the burden’ of the illness. The above recommendations suggest ways in which women can be safeguarded to ensure a child’s illness does not increase their vulnerability. Although men are involved in financial provision both for childcare and to seek treatment during illness, we observed that they are largely absent in all other aspects related to the child illness. This is not to suggest that men were unconcerned about their child’s welfare, rather it is a demonstration of how societal gendered norms, expectations and responsibilities influence broader life aspects. In fact, during the period of hospital admission we often observed these fathers coming to visit their wives and children in the wards and engaging with health workers to get an update on their children’s health indicating genuine concern. As flagged above, there is current planned work in the same study areas to use participatory approaches to explore and understand - from the perspective of men - barriers and facilitators to male engagement in child health. Men play a crucial role in the overall family set up and it is therefore important to actively engage them in matters of child health for overall better outcomes. Finally, given the intersections of gender with other social categories such as age and professional and socio-economic status as highlighted in this study, future work should consider applying a deliberate intersectional lens to develop more nuanced understanding of these intersections and related power structures, and how that interacts with child illness and treatment-seeking.

## Data Availability

Given the sensitive nature of qualitative data, the raw data/datasets generated as part of this work are not publicly available to safeguard participant confidentiality and anonymity. Requests for data related to this work can be channelled through the KEMRI-Wellcome Trust Research Programme Data Governance Committee.

## Abbreviations

APHRC: Africa Population Health Research Centre
CHAIN: Childhood Acute Illness & Nutrition Network
HH: Household
IC: Index child
LMICs: Low- and middle-income countries
MW: Moderate wasting
NGO: Non-governmental organisation
NW: No wasting
SWK: Severe wasting or Kwashiorkor
